# A Change Detection Method for Remote Sensing Images Based on Coupled Dictionary and Deep Learning

**DOI:** 10.1155/2022/3404858

**Published:** 2022-01-17

**Authors:** Weiwei Yang, Haifeng Song, Lei Du, Songsong Dai, Yingying Xu

**Affiliations:** School of Electronics and Information Engineering, Taizhou University, Taizhou 318000, Zhejiang, China

## Abstract

With the rapid development of remote sensing technology, change detection (CD) methods based on remote sensing images have been widely used in land resource planning, disaster monitoring, and urban expansion, among other fields. The purpose of CD is to accurately identify changes on the Earth's surface. However, most CD methods focus on changes between the pixels of multitemporal remote sensing image pairs while ignoring the coupled relationships between them. This often leads to uncertainty about edge pixels with regard to changing objects and misclassification of small objects. To solve these problems, we propose a CD method for remote sensing images that uses a coupled dictionary and deep learning. The proposed method realizes the spatial-temporal modeling and correlation of multitemporal remote sensing images through a coupled dictionary learning module and ensures the transferability of reconstruction coefficients between multisource image blocks. In addition, we constructed a differential feature discriminant network to calculate the dissimilarity probability for the change area. A new loss function that considers true/false discrimination loss, classification loss, and cross-entropy loss is proposed. The most discriminating features can be extracted and used for CD. The performance of the proposed method was verified on two well-known CD datasets. Extensive experimental results show that the proposed method is superior to other methods in terms of precision, recall, *F*_1_-score, IoU, and OA.

## 1. Introduction

In 1858, the first remote sensing image was taken over Paris, France. Human's cognition of the Earth has entered a new stage from local to large-scale surface observation as a result of remote sensing technology on various platforms. In recent years, with the rapid development of sensor and information technology, it has become possible to acquire and store large amounts of remote sensing images. Remote sensing images provide extensive Earth observational data that are characterized by high precision, wide range, and short period, offering important data support for the military, science, agriculture, environment, resources, transportation, and urban management [1]. Researching the surface ecological environment using these remote sensing images has great societal and scientific significance [2].

Remote sensing image change detection (CD) is an important application of remote sensing image interpretation that aims to compare and analyze two (or more) remote sensing images taken at different times in the same region and obtain change information about the ground object. This technology has been widely used in land planning [3], urban expansion [4], disaster assessment [5], agricultural investigation [6], scene classification [7], and ecological environment monitoring [8], among other fields. Traditional artificial visual interpretation methods mainly detect changes in the obtained remote sensing images using the visual interpretation experience, which has low efficiency and is easily affected by subjective conditions. Therefore, such methods cannot meet current needs for dynamic monitoring of the environment. With the development of remote sensing image CD technology, although new CD methods continue to emerge, many new problems and challenges have arisen due to improvements in imaging ability and different properties of sensors. The main problems and challenges are as follows. First, the continuous improvement of spatial and spectral resolution of remote sensing images offers abundant ground object information, which significantly increases redundancy and noise. Second, with the continuous development of sensor technology, various new data sources are emerging. This poses a new challenge to the universality of existing remote sensing image processing methods. How to accurately and quickly extract useful information from massive datasets for specific tasks has become an urgent problem. Third, the applications for remote sensing images continuously involve more and more fields. Thus, a single model can no longer complete most of the complex tasks required. Therefore, the existing image classification and CD models must start with a practical application and fully combine the advanced technologies in various fields to meet the needs of different industries. However, for a variety of complex tasks, it is difficult for human beings to obtain an effective and comprehensive model due to limited prior knowledge of the task. Identifying new methods to solve the aforementioned problems has increasingly attracted researchers' attention.

With the rapid development of computer theory and technology, artificial intelligence technology has appeared in image segmentation, image classification, CD, and other related research areas. In particular, the recent emergence of deep learning methods as a powerful driving force of artificial intelligence technology has greatly stimulated the enthusiasm of various research fields to utilize deep learning to solve existing problems. The outstanding deep learning model UNet is widely used in segmentation and achieved good performance in multiobject [9, 10] and multiscale [11] remote sensing image segmentation. In respect of remote sensing image classification, the DRL-GAN is proposed by literature [12]; the network can not only enhance the image representation ability, but also identify tiny objects from remote sensing images. As for remote sensing image translation, the attention mechanism is introduced to the generative adversarial network architecture, and the SPA-GAN model is proposed by literature [13]. With regard to remote sensing image dehazing, considering the CNN's excellent performance in image dehazing, the FD-GAN is proposed by literature [14]; the model can produce more realistic and natural images with less color distortion and fewer artifacts. Concerning remote sensing image object detection, an architecture with ESRGAN, EEN, and detection network is proposed by literature [15]; the quality of remote sensing images is enhanced by using EESRGAN, and the detection performance is improved by backpropagating the loss into the EESRGAN. From the above achievement, we can discover that the rich hierarchical architecture of deep learning networks is conducive to adopting shallow to deep approaches. The target task is analyzed in depth and thoroughly, and the understanding of high-level and high-dimensional features is gradually formed through low-level information analysis. The strong feature learning and expression abilities of deep learning models can be used to realize automatic robust learning if the model is able to effectively utilize a large amount of existing information. A large number of training models based on deep learning methods within the category of supervised learning can provide sufficient reference for massive multisource datasets, greatly improving the utilization of data and the timeliness of a variety of remote sensing tasks, including CD.

At present, deep learning methods have been used to carry out a great deal of research in the field of remote sensing image target extraction. The theory underlying such methods should also be applicable to the field of CD. High-level semantic features such as spectrum, space, and texture can be extracted from multitemporal images using deep learning to establish a nonlinear relationship between ground object features in multitemporal images. Remote sensing CD models based on deep learning are different from traditional remote sensing CD models in that deep learning can identify the features of data through representation learning, which can reduce the heavy dependence on manual work during feature extraction in remote sensing image CD. Therefore, researching remote sensing image CD methods based on deep learning can not only improve the accuracy of remote sensing image CD, but also offer a new automatic and intelligent processing method for realizing current application requirements.

The main contributions of the paper can be summarized as follows:

1. In this paper, a change detection framework based on coupled dictionary learning is proposed to map multisource and multitemporal data from low-dimensional space to high-dimensional space and then estimate its change.
2. A differential feature extraction network is proposed to construct a new reconstruction loss by integrating true-false discrimination loss, classification loss, and cross-entropy loss, which is conducive to training the coupled dictionary with stronger representation ability.
3. The multisource remote sensing image change detection is realized by combining the advantages of model driven method (coupled dictionary learning) and data driven method (differential feature extraction network), and data from different observation spaces can be modeled uniformly.

The rest of this paper is organized in four sections.  Section 2 is a description for related works of the change detection task we are dealing with.  Section 3 provides details on the proposed model.  Section 4 deals with the experimental results and discussion. Finally, Section 5 summarizes the observations and points out some probable future works to complete this paper.

## 2. Related Works

CD refers to the process of observing the same object on the Earth's surface at different times to determine its different states. The task of CD is to answer three questions about object change: (1) Has it changed? (2) Where has the change occurred? (3) What has changed? With the increasing resolution of sensors, the acquisition period of remote sensing images has gradually shortened, and a large number of CD methods based on remote sensing images have been proposed.

In recent years, the emergence of deep learning technology has provided new concepts for CD in remote sensing images and shown strong ability to solve the problems associated with traditional methods. First, compared with traditional methods, deep learning methods do not require a large amount of existing knowledge. Second, no manual intervention is required during the training process, which greatly improves the automation of remote sensing image CD. Third, since deep neural networks have strong learning and feature expression abilities, they are more suitable for processing high-dimensional data than traditional methods. Therefore, remote sensing image CD methods based on deep learning methods are attracting increasing attention. Many deep learning methods, such as NN [16], FCN [17], DenseNet [18], UNet [19], DeepLab [20], and SegNet [21], have been introduced for remote sensing image applications and have achieved extremely high accuracy. Some research results have even approached or attained the accuracy of human vision systems. Previous literature [22] has used an end-to-end recurrent neural network with a long short-term memory (LSTM) network to learn change rules of land cover. Literature [23] trained a deep belief network to classify changing and unchanging regions in SAR images, while literature [24] proposed an end-to-end two-dimensional convolutional neural network for hyperspectral image CD. Other studies [25, 26] have researched the use of generative adversarial networks to detect the change areas of multispectral remote sensing images and other cross-domain remote sensing images. Literature [27] proposed a deep Siamese convolutional network (DSCN) to detect changing regions with contrast loss in images. A symmetric convolutional coupled network (SCCN) based on a DSCN was proposed in literature [28] to detect changes between optical and SAR images. In sum, existing CD methods based on deep learning can autonomously learn high-dimensional features of the changed region without manual intervention and perform CD according to the truth value.

Most CD methods analyze multitemporal remote sensing data obtained by the same sensor or same type of sensor. However, with the development of remote sensing imaging technology, there are more and more multisource remote sensing data covering the same area. The imaging mechanisms and results of these multisource data are quite different, making it difficult to detect changes using traditional CD technology. Although there may be major differences in multisource data, they ultimately constitute different descriptions of the same scene. The imaging makes comparisons difficult in the original low-dimensional space, but having the same ground object makes multisource data comparable in high-dimensional space. On the other hand, although rich data are provided by sensors, these data are often the result of a combination of factors. Therefore, it is necessary to identify the key relevant information to explain and assist in decision-making. In recent years, sparse representation has increasingly been used in remote sensing image CD and data fusion. In 2021, in order to solve the problem that the accuracy of CD is easily affected by speckle noise, a method based on sparse representation and capsule network was proposed in literature [29]. First, sparse features of difference images are extracted using SR. Second, a lightweight capsule network (L-CapsNet) is constructed to mine the spatial relationships between features. Finally, a change map (CM) is generated. Experimental results show that this method can reduce the influence of speckle noise and obtain stronger robust features. An image fusion method based on sparse representation has also been proposed in the literature [30]. The method is based on the assumption that NDVI and HRVI with different resolutions have the same sparse coefficients in a particular dictionary. The application of this method in multisource NDVI CD was verified using GF-1 and GF-2 satellite datasets.

As mentioned above, multisource image pairs are different descriptions of the same object. The data representation and correlation in different observation space constitute a challenging task. Due to the difference of imaging mechanism, multisource images have completely different imaging results. This encouraged us to try to learn two independent and coupled dictionaries to relate different observation spaces. The dictionary can be coupled by forcing multiple image block pairs to share the same sparse representation. Inspired by the above idea, a coupled dictionary learning method is proposed for multisource image change detection. The goal of coupled dictionary learning is to match the elements from different observation spaces, so that they have one-to-one correspondence in the same position, and realize the mapping of multisource data. Through coupling dictionary learning, invariant image block pairs tend to have lower reconstruction errors, while variable image block pairs have higher reconstruction errors. This can suppress the unchanged area, highlight the changed area, and obtain a robust change map. However, the randomly selected training sample may contain some changing training samples, and these changing samples may interfere with the learning of the coupled dictionary. Therefore, we design a new loss function to automatically select training samples with low reconstruction errors and exclude training samples with changes in the training set, so as to build a more robust coupled dictionary. Finally, the multisource data is mapped to a high-dimensional feature space to enhance the variation of multisource data.

## 3. Proposed Method

Firstly, we give a general introduction to the proposed model in this paper; the input of the model is the original remote sensing images, and the output of the model is the CD results of the corresponding pixel. Then, the data preprocessing method, coupled dictionary learning method, and differential feature extraction network are introduced in detail. At the same time, a loss function for CD is introduced.

### 3.1. Overview of the Proposed Method

**Figure 1:**
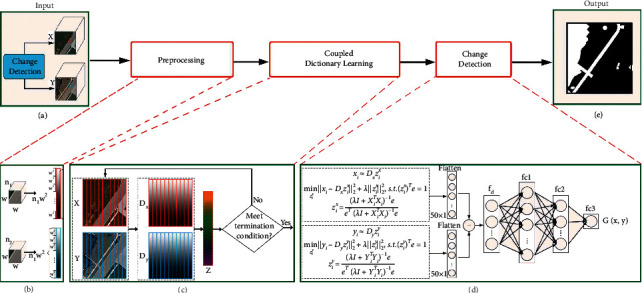
Overview of the proposed method.

As shown in Figure 1, the method proposed in this paper consists of five steps: input, preprocessing, coupled dictionary learning, CD, and output. First, the input consists of multitemporal and multisource remote sensing image data. Second, in the preprocessing step, local image blocks of two different periods data obtained by different sensors on the same scene are expanded into vector representations. Third, each image block pair is stacked as a training sample for coupled dictionary learning and forced to share the same reconstruction coefficient. After the coupled dictionary is learned, it can be determined whether the current coupled dictionary meets the termination condition. If not, the training sample with low reconstruction error is chosen as the next generation of the training set to learn the dictionary pairs with higher coupled degrees (and, if necessary, the process is repeated). Fourth, based on the coupled dictionary learned in the second step, the reconstruction coefficient of each image block pair is calculated and input into the difference feature extraction network to estimate the change degree. Finally, the final binary CD result is produced as output.

### 3.2. Data Preprocessing

The concept of sparsity first appeared in the 1970s in the context of the efficient coding hypothesis in neurobiology. Today, this idea is widely used in the field of image processing. The following preprocessing should be carried out before attempting remote sensing shadow CD.

Let *X*={*x*_*i*_}_*i*=1_^*N*^ and *Y*={*y*_*i*_}_*i*=1_^*N*^ represent *n* pairs of image blocks. Each pair (*x*_*i*_, *y*_*i*_) is randomly sampled from two multisource remote sensing images taken at different times in the same geographical location. In the proposed method, two image blocks (*x*_*i*_, *y*_*i*_) at the same position are regarded as a pair of image blocks. As shown in Figure 1(b), an image block of size *w* × *w* × *n*_*t*_(*t*=1,2) can be represented as *R*^*m*_*t*_^, where *m*_*t*_=*n*_*t*_*w*^2^. *x*_*i*_ and *y*_*i*_ can be regarded as column vectors with dimensions *m*_1_ and *m*_2_, respectively.

### 3.3. Coupled Dictionary Learning

The typical problems related to sparse representation are expressed as in the following equation:

(1) $$\begin{array}{c} \underset{D,\alpha_{i}}{\min}\sum_{i=1}^{N}{\left\|x_{i}-D\alpha_{i}\right\|}_{2}^{2}+\lambda {\left\|\alpha_{i}\right\|}_{1},\\ \text{s.t.}{\left\|d_{j}\right\|}^{2}⩽1,j=1,2,\ldots ,k. \end{array}$$

The *i*^th^ image block is represented as *x*_*i*_; *D*=[*d*_1_, *d*_2_,…, *d*_*n*_] ∈ *R*^*m*×*d*^ represents the dictionary corresponding to the image; *α*_*i*_ represents the sparse coefficient, which is obtained by sparse representation of the *i*^th^ image block *x*_*i*_ according to dictionary *D*; *λ* is the regular parameter; and *λ*‖*α*_*i*_‖_1_ ensures the sparsity of vector *α*_*i*_. Finally, the constraint on ‖*d*_*i*_^2^‖^2^ ensures that each dictionary atom is homogeneously distributed in order to avoid too-large values that will increase the time necessary for optimization.

The coupled dictionary learning method for remote sensing images proposed in this paper is an extension of the above methods. Let *D*_*x*_=[*d*_1_^*x*^, *d*_2_^*x*^,…, *d*_*n*_^*x*^] ∈ *R*^*m*_1_×*d*^ and *D*_*y*_=[*d*_1_^*y*^, *d*_2_^*y*^,…, *d*_*n*_^*y*^] ∈ *R*^*m*_2_×*d*^ contain *d* elements (respectively, *d*_*i*_^*x*^ ∈ *R*^*m*_1_^ and *d*_*i*_^*y*^ ∈ *R*^*m*_2_^). The two images are represented by *X*=*x*_*i*_ _*i*=1_^*n*^ and *Y*=*y*_*i*_ _*i*=1_^*n*^. Image blocks *x*_*i*_ and *y*_*i*_ share the same reconstruction coefficient. The sparse representation of the two images can be combined into an equation as follows:

(2) $$\begin{array}{c} \underset{D_{x},D_{y},\alpha_{i}}{\min}\sum_{i=1}^{N}\left\{\frac{1}{2}{\left\|x_{i}-D_{x}\alpha_{i}\right\|}_{2}^{2}+\lambda {\left\|\alpha_{i}\right\|}_{1}\right\}+\left\{\frac{1}{2}{\left\|y_{i}-D_{y}\alpha_{i}\right\|}_{2}^{2}+\lambda {\left\|\alpha_{i}\right\|}_{1}\right\},\\ \text{s.t.}{\left\|d_{i}^{2}\right\|}^{2}⩽1,i=1,2,\ldots ,k. \end{array}$$

Let $\bar{X}=\left[\begin{array}{c} x_{i}\\ y_{i} \end{array}\right]$, $\bar{D}=\left[\begin{array}{c} D_{x}\\ D_{y} \end{array}\right]$; (2) can be written as follows:

(3) $$\begin{array}{c} \underset{D,\alpha_{i}}{\min}\sum_{i=1}^{N}{\left\|\bar{X}-\bar{D}\alpha_{i}\right\|}_{2}^{2}+\lambda {\left\|\alpha_{i}\right\|}_{1},\\ \text{s.t.}{\left\|\bar{d_{j}}\right\|}^{2}⩽1,j=1,2,\ldots ,k, \end{array}$$

where $\bar{d_{j}}$ is the *i*^th^ element of the coupled dictionary $\bar{D}$.

After the above combination, the complex equations are simplified into sparse representation objective functions, and the corresponding optimization process is also simplified. Equation (3) is not a convex function, which is not convenient for optimization. However, when one of the values is fixed, the above equation becomes a convex function that can be optimized. Therefore, the alternate iteration method is used to optimize it. The specific calculation method is as follows:

(1) Optimizing coupled sparse coefficient *α*_*i*_. According to the above analysis, dictionary $\bar{D}$ must be fixed before coupled sparse coefficient *α*_*i*_ is optimized. For the first iteration, $\bar{D}$ is unknown. The dictionary is initialized by randomly selecting data in the training set or randomly generating a matrix. After dictionary $\bar{D}$ is determined, the objective function becomes a sparse coding problem, and the sparse coefficients are calculated according to the categories. When the *i*^th^ sparse coefficient *α*_*i*_ is calculated, the remaining sparse coefficients that do not belong to the *α*_*i*_(*i* ≠ *j*) are fixed. The following equation can be used to solve the sparse coefficient *α*_*i*_:
  (4) $$\begin{array}{c} \underset{\alpha_{i}}{\min}{\left\|\bar{x_{i}}-\bar{D_{x}}\alpha_{i}\right\|}_{2}^{2}+{\left\|\bar{x_{i}}-\bar{D_{x}}\alpha_{i}^{i}\right\|}_{2}^{2}+\sum_{j=1,j\neq i}^{c}{\left\|\bar{D_{j}}\alpha_{i}^{j}\right\|}_{2}^{2}+\lambda {\left\|\alpha_{i}\right\|}_{1}. \end{array}$$
The iterative projection method [31] is used to solve (4).
(2) Optimizing dictionary $\bar{D}$. In this optimization process, the sparse coefficient *α*_*i*_ is a known condition. The method of updating the dictionary is similar to the previous procedure. When the dictionary $\bar{D_{i}}$ belonging to the *i*^th^ category is computed, the rest of the dictionaries $\bar{D_{j}},\left(j\neq i\right)$ are fixed. The objective function can be simplified to the following equation:
  (5) $$\begin{array}{c} \underset{\bar{D_{i}}}{\min}{\left\|\bar{x_{i}}-\bar{D_{x}}\alpha_{i}-\sum_{j=1,j\neq i}^{c}\bar{D_{x}}\alpha_{i}\right\|}_{2}^{2}+{\left\|\bar{x_{i}}-\bar{D_{x}}\alpha_{i}^{i}\right\|}_{2}^{2}+\sum_{j=1,j\neq i}^{c}{\left\|\bar{D_{j}}\alpha_{j}^{i}\right\|}_{2}^{2}. \end{array}$$
$\bar{x_{i}}$ represents the sparse coefficient obtained by sparse representation of training image set $\bar{X}$ on dictionary $\bar{D}$, which belongs to the *i*^th^ category.

### 3.4. Differential Feature Extraction Network

After obtaining the coupled dictionary *D*_*x*_ and *D*_*y*_, the multisource remote sensing image data is mapped to the high-dimensional feature space. In this space, changing image blocks will have different reconstruction coefficients, while unchanging image blocks will have similar reconstruction coefficients.

The change degree of each image block pair can be measured by comparing the reconstruction coefficients *α*_*i*_^*x*^ and *α*_*i*_^*y*^ of the *i*^th^ pair of images *x*_*i*_ and *y*_*i*_ with their coupled dictionaries *D*_*x*_ and *D*_*y*_. The calculation method for *α*_*i*_^*x*^ and *α*_*i*_^*y*^ is shown in (6), (7), and (8).

(6) $$\begin{array}{c} \left\{\begin{array}{c} x_{i}\approx D_{x}\alpha_{i}^{x},\\ y_{i}\approx D_{x}\alpha_{i}^{y}. \end{array}\right) \end{array}$$

*α*_*i*_^*x*^ and *α*_*i*_^*y*^ can be solved as in

(7) $$\begin{array}{c} \left\{\begin{array}{c} \underset{z_{i}^{x}}{\min}{\left\|x_{i}-D_{x}\alpha_{i}^{x}\right\|}_{2}^{2}+\lambda {\left\|\alpha_{i}^{x}\right\|}_{2}^{2},\\ \underset{z_{i}^{y}}{\min}{\left\|y_{i}-D_{y}\alpha_{i}^{y}\right\|}_{2}^{2}+\lambda {\left\|\alpha_{i}^{y}\right\|}_{2}^{2}. \end{array}\right) \end{array}$$

*λ* is the regular parameter. Equation (7) can be solved as in

(8) $$\begin{array}{c} \left\{\begin{array}{c} \alpha_{i}^{x}=\frac{{\left(\lambda I+X_{i}^{T}X_{i}\right)}^{-}1e}{e^{T}{\left(\lambda I+X_{i}^{T}X_{i}\right)}^{-}1e},\\ \alpha_{i}^{y}=\frac{{\left(\lambda I+Y_{i}^{T}Y_{i}\right)}^{-}1e}{e^{T}{\left(\lambda I+Y_{i}^{T}Y_{i}\right)}^{-}1e}. \end{array}\right) \end{array}$$

*e* is a vector of all 1s in *R*^*d*^, *X*_*i*_=[*d*_1_^*x*^ − *x*_*i*_, *d*_2_^*x*^ − *x*_*i*_,…, *d*_*d*_^*x*^ − *x*_*i*_] ∈ *R*_1_^*m*^ × *d*, *Y*_*i*_=[*d*_1_^*y*^ − *y*_*i*_, *d*_2_^*y*^ − *y*_*i*_,…, *d*_*d*_^*y*^ − *y*_*i*_] ∈ *R*_2_^*m*^ × *d*, and *I* is the unit matrix of *d* × *d*.

Since the goal of CD is to detect the differences between two images taken at different times, the difference feature vectors are calculated after the feature vectors *α*_*i*_^*x*^ and *α*_*i*_^*y*^ of the two images are obtained using the above method. The difference feature vector *α*_*d*_ is calculated as in

(9) $$\begin{array}{c} \alpha_{d}=\alpha_{i}^{x}-\alpha_{i}^{y}. \end{array}$$

In order to learn the dissimilarity probability between two images from the difference feature vectors, a difference feature extraction network is designed after the difference feature vectors are obtained. The network consists of three fully connected layers, *f*_1_, *f*_2_, *f*_3_, as shown in Figure 1(d). The value of its hidden layer can be calculated using the following equation:

(10) $$\begin{array}{c} h^{\left(k\right)}=\text{ReLu}\left(W^{\left(k\right)}h^{\left(k-1\right)}+b^{\left(k\right)}\right). \end{array}$$

*h*^(*k*)^ and *h*^(*k* − 1)^ are the output matrices of the *k* ^th^ and (*k* − 1)^th^ layers. *W*_*k*_ is the weight that is input to the hidden layer, *b*^(*k*)^ is the bias, and because we need is a binary value of 0 or 1, the ReLU(·) is selected as the activation function. When the information is transmitted to the last fully connected layer *fc*3, a value ranging from 0 to 1 is output, indicating the probability of dissimilarity.

For the above differential feature discriminant network, it is expected that the generated dissimilarity probability can be judged as the correct category label and that the obtained dissimilarity probability will be large for the changing category and small for the unchanging category. Therefore, cross-entropy loss is added to its loss function. Its loss function consists of three parts, as shown in the following equation:

(11) $$\begin{array}{c} L_{G}=\min\left\{L_{\text{adv}}+L_{\text{cls}}+L_{E}\right\}. \end{array}$$

*L*_adv_ represents true/false loss, *L*_cls_ represents category loss, and *L*_*E*_ represents cross-entropy loss. They are calculated as in the following equations:

(12) $$\begin{array}{c} L_{\text{adv}}=E_{X,Y∼p\left(X,Y\right)}\left[\log\left(1-D\left(G\left(X,Y\right)\right)\right)\right], \end{array}$$

(13) $$\begin{array}{c} L_{cls}=E\left[\log\left(C=c|y_{\text{fake}}\right)\right], \end{array}$$

(14) $$\begin{array}{c} L_{E}=\frac{1}{N}\left[\sum_{i=1}^{N}y^{i}y_{\text{fake}}^{i}+\left(1-y^{i}\right)\log\left(1-y_{\text{fake}}^{i}\right)\right]. \end{array}$$

*N* is the number of training samples, and the value of *y*^*i*^ is 0 or 1, which indicates the label of the input neighborhood image block pairs. 1 indicates that they have changed, and 0 indicates that they have not changed. *y*_fake_^*i*^ represents the probability of dissimilarity of input neighborhood image block pairs.

For the optimize algorithm, SGD algorithm is a famous parameter optimization method proposed, which is widely used in model parameter optimization of deep learning. However, its disadvantage is that it has a slow descent speed and may oscillate near the minimum value [32]. Therefore, SGD method based on first-order momentum is proposed [33]. This method updates each parameter with the same learning rate. However, deep neural network often contains a large number of parameters. For the parameters which are updated frequently, the model has accumulated a lot of knowledge about them; we hope that they are affected by a single sample, so the learning rate should be slower. However, for parameters that are occasionally updated, the model has little accumulated knowledge, and we hope that more knowledge can be learned from each random sample, so the learning rate should be higher. Therefore, an optimization method named AdaGrad based on second-order momentum is proposed [34]. This method performs well for sparse data. However, it may reduce the learning rate monotonously to zero, thus ending the training process prematurely. Adam [35] optimization method solves the above problems completely. This method integrates first-order momentum and second-order momentum, and the coefficient is used to control the first-order momentum and second-order momentum, which is more conducive to large-scale remote sensing image optimization. Therefore, the Adam algorithm is used to optimize loss function *L*_*G*_. During the training process, all parameters of the network are fixed first, and they are updated. When *L*_*G*_ training tends toward stability, this indicates that the network has reached a convergence state and the training process can be stopped.

## 4. Experiment and Discussion

### 4.1. Dataset

**Figure 2:**
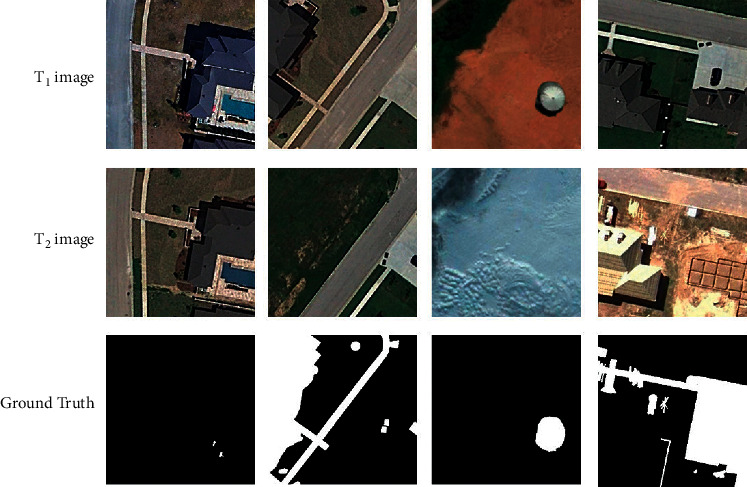
The samples of CDD.

The first dataset is the CDD [36], shown in Figure 2. It consists of seven pairs of images with 4725 × 2700 resolution obtained from Google Earth. A total of 16,000 image pairs (10,000 in the training set, 3,000 in the test set, and 3,000 in the validation set) with a spatial resolution of 3 − 100 cm were collected from the seven pairs of images.

**Figure 3:**
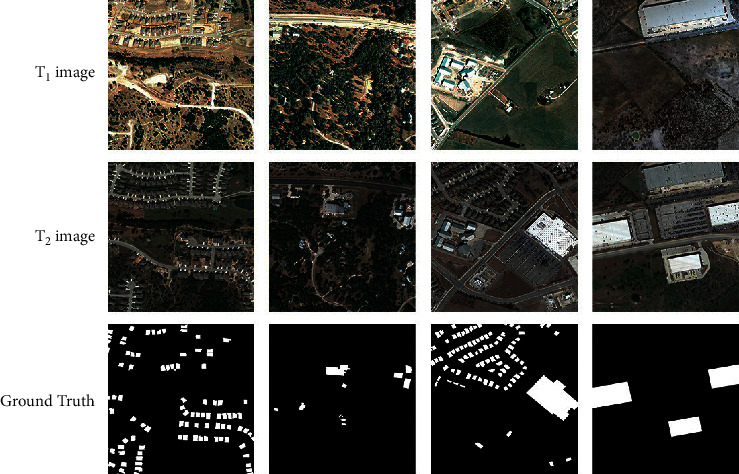
The samples of LEVIR-CD dataset.

The second dataset is the LEVIR-CD dataset [37], shown in Figure 3. The dataset contains 637 pairs of CD 1024 × 1024 images with a spatial resolution of 0.5 m. The images were collected from 20 different locations in Texas between 2002 and 2018. This dataset helps train more efficient CD models while focusing on interesting changes and reducing the impact of other irrelevant changes on the model.

### 4.2. Evaluation Metrics

For CD, it is necessary to prove the effectiveness and robustness of the proposed method using quantitative analysis. For performance evaluation purposes, CD results are usually presented as binary images, wherein white pixels represent changed pixels and black pixels represent unchanged pixels. In this experiment, precision, recall, *F*_1_-score, OA, and IoU were used to evaluate the proposed methods. The calculation methods are shown in the following equations:

(15) $$\begin{array}{c} \text{precision}=\frac{\text{TP}}{\text{TP}+\text{FP}}, \end{array}$$

(16) $$\begin{array}{c} \text{recall}=\frac{\text{TP}}{\text{TP}+\text{FN}}, \end{array}$$

(17) $$\begin{array}{c} F1-\text{score}=\frac{2\times \text{precision}\times \text{recall}}{\text{precision}+\text{recall}}, \end{array}$$

(18) $$\begin{array}{c} OA=\frac{\text{TP}+\text{TN}}{\text{TP}+\text{TN}+\text{FP}+\text{FN}}, \end{array}$$

(19) $$\begin{array}{c} \text{IoU}=\frac{\left(\text{TP}/\left(\text{TP}+\text{FP}+\text{FN}\right)+\text{TN}/\left(\text{TN}+\text{FN}+\text{FP}\right)\right)}{2}. \end{array}$$

### 4.3. Experimental Setting

For the above two remote sensing image CD datasets, experiments were carried out to verify the accuracy and effectiveness of the proposed method. For the CDD, the training dataset contained 10,000 images, the test dataset and validation dataset contained 3,000 images each, and the resolution of each image was 256 × 256. For the LEVIR-CD dataset, the training dataset contained 445 images, the test dataset contained 128 images, the validation dataset contained 64 images, and the resolution of each image was 1024 × 1024.

The CPU of the computer used in this experiment was an Intel Xeon Silver 4212, 2.2 GHz, with a memory size of 512 GB. The GPU was an NVIDIA Tesla V100, 32 GB. The CUDA version was 10.2. The operating system was Microsoft Windows Server 2016 Datacenter. The *Python* version was 3.6.10, and the deep learning framework was PyTorch version 1.9.0.

During the training process, due to the low resolution of the CDD, its batch size was set to 16, while due to the high resolution of the LEVIR-CD dataset, its batch size was set to 2 in consideration of the capacity of the system. The initial learning rate was set to 1e-3 and was adjusted automatically by the system according to the training situation. The number of epochs was set to 100. The K-SVD algorithm was used to learn the coupled dictionary. The differential feature extraction network was composed of three fully connected layers (*fc*_1_, *fc*_2_,  and *fc*_3_). The range of the numbers in the hidden layer of the differential feature extraction network was determined by empirical formulas and Kolmogorov theorem [38]. The number of nodes was set to 150, 100, and 1, respectively.

### 4.4. Ablation Analysis

Ablation analysis was used to illustrate the contribution of the proposed method's component. We assessed the contribution of the component to the model by comparing the model that contains it with the one that does not. Instead of subtracting, we gradually added modules such as coupled dictionary learning (CDL), differential feature extraction (DFE), and *L*_*G*_ loss function (*L*) onto our proposed baseline network to verify its performance. Compared with LEVID-CD dataset, CDD is a large-scale remote sensing dataset. Therefore, the CDD is used for ablation analysis.

Table 1 shows the results of the ablation experiment. The last row in the table represents the performance of our proposed model. The first row represents the performance of the baseline model (sparse representation). The next row in Table 1 indicates that deploying the coupled dictionary learning module, differential feature extraction, and *L*_*G*_ loss function to the baseline model leads to a significant improvement in all the metrics. Adding the coupled dictionary learning module to the model results in a slight improvement in all the metrics except the recall rate. The *F*_1_-score is improved due to the addition of differential feature extraction. We then introduce the *L*_*G*_ loss function, which raises all the metrics except slightly. This variation can be attributed to *L*_*G*_ loss function, which enhances the representation ability for the proposed method.

### 4.5. Results and Discussion

To evaluate the performance and effectiveness of the proposed method, we compared it with several classical methods.

**Figure 4:**
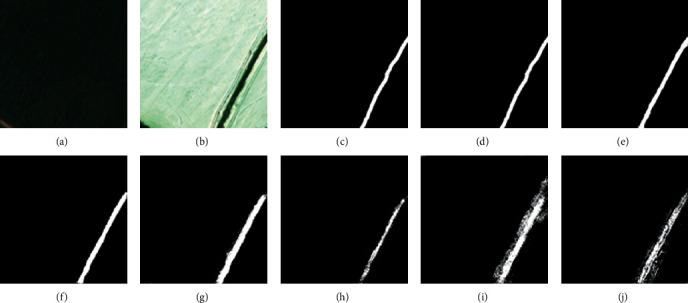
Qualitative comparison of different approaches on CDD for road change. (a) T1 image (#0257). (b) T2 image (#0257). (c) Reference change map. (d) Ours. (e) CD-UNet++. (f) CDNet. (g) FC-Siam-Diff. (h) FC-Siam-Conc. (i) FCN-PP. (j) FC-EF.

**Figure 5:**
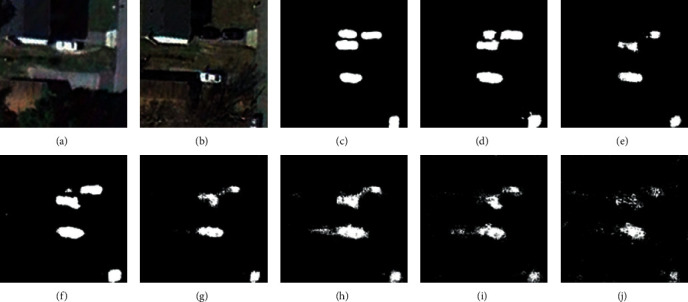
Qualitative comparison of different approaches on CDD for car change. (a) T1 image (#0327). (b) T2 image (#0327). (c) Reference change map. (d) Ours. (e) CD-UNet++. (f) CDNet. (g) FC-Siam-Diff. (h) FC-Siam-Conc. (i) FCN-PP. (j) FC-EF.

**Figure 6:**
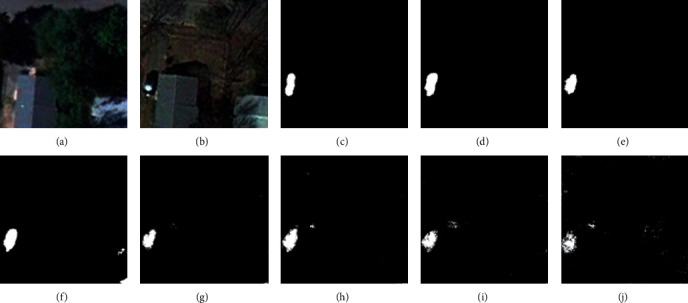
Qualitative comparison of different approaches on CDD for single-object change. (a) T1 image (#0628). (b) T2 image (#0628). (c) Reference change map. (d) Ours. (e) CD-UNet++. (f) CDNet. (g) FC-Siam-Diff. (h) FC-Siam-Conc. (i) FCN-PP. (j) FC-EF.

**Figure 7:**
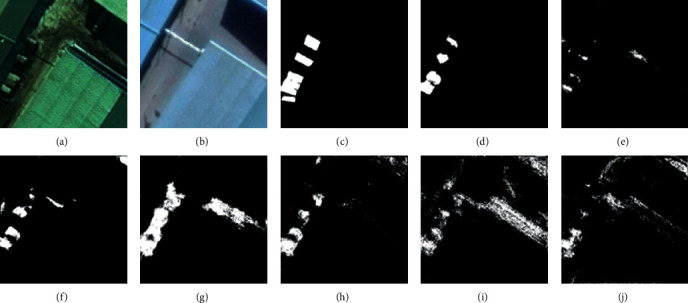
Qualitative comparison of different approaches on CDD for multiple-object change. (a) T1 image (#0283). (b) T2 image (#0283). (c) Reference change map. (d) Ours. (e) CD-UNet++. (f) CDNet. (g) FC-Siam-Diff. (h) FC-Siam-Conc. (i) FCN-PP. (j) FC-EF.

For the CDD, we used seven state-of-the-art (SOTA) methods: FC-EF [39], FCN-PP [40], FC-Siam-Conc [39], FC-Siam-Diff [39], CDNet [41], CD-UNet++ [42], and GANs [43]. The experimental results and visual comparisons are shown in Table 2 (some of the experimental results were obtained from [44]) and Figures 4to 7.

In this experiment, four typical change areas, road, car, single object, and multiple objects, were selected to evaluate the performance of the proposed method. As can be seen from Figures 4to 7, the proposed method consistently achieved the best performance. The boundary of the proposed method's CM is clearer than those of the other methods, and the misclassification area of the proposed method is smaller than those of the other methods. In addition, the change maps obtained by the proposed method are almost consistent with the reference CM.

Meanwhile, the quantitative results in terms of the five quantitative evaluation metrics listed in Table 2 show that the proposed method achieved excellent performance in terms of precision, recall, *F*_1_-score, IoU, and OA. The OA of the proposed method was 0.883, which is 0.02 higher than that of the GANs method (which had the best precision among the seven SOTA CD methods). The accuracy value was also significantly improved compared with the other methods.

**Figure 8:**
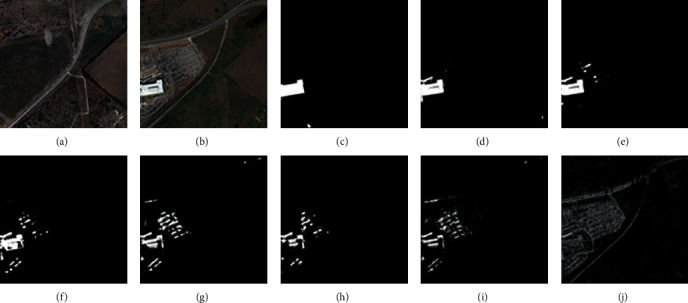
Qualitative comparison of different approaches on LEVID-CD dataset for single-object change. (a) T1 image (#0104). (b) T2 image (#0104). (c) Reference change map. (d) Ours. (e) IFN. (f) DASNet. (g) CD-UNet++. (h) FC-Siam-Conc. (i) FC-Siam-Diff. (j) FC-EF.

**Figure 9:**
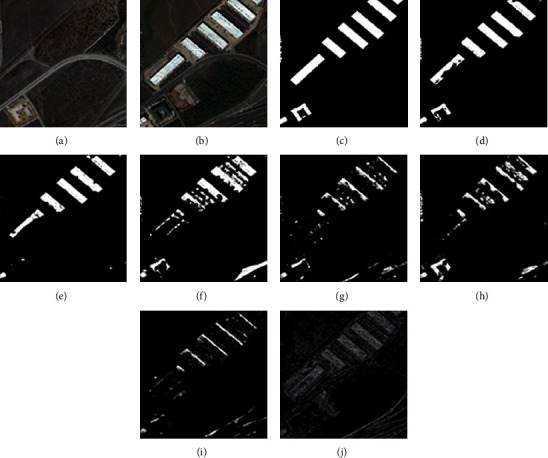
Qualitative comparison of different approaches on LEVID-CD dataset for more-object change. (a) T1 image (#0102). (b) T2 image (#0102). (c) Reference change map. (d) Ours. (e) IFN. (f) DASNet. (g) CD-UNet++. (h) FC-Siam-Conc. (i) FC-Siam-Diff. (j) FC-EF.

**Figure 10:**
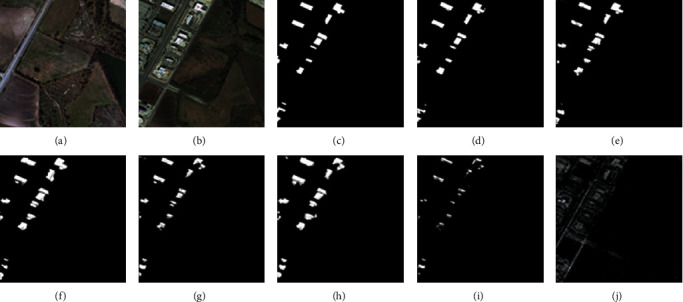
Qualitative comparison of different approaches on LEVID-CD dataset for much-more-object change. (a) T1 image (#0054). (b) T2 image (#0054). (c) Reference change map. (d) Ours. (e) IFN. (f) DASNet. (g) CD-UNet++. (h) FC-Siam-Conc. (i) FC-Siam-Diff. (j) FC-EF.

**Figure 11:**
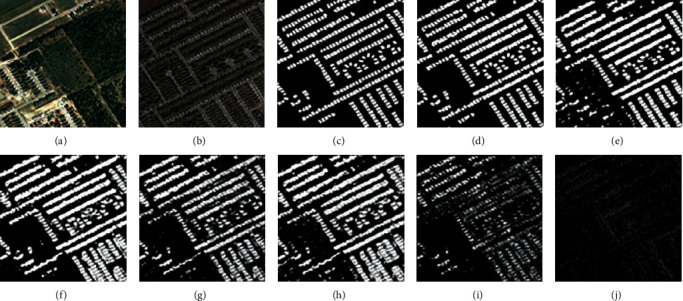
Qualitative comparison of different approaches on LEVID-CD dataset for dense-object change. (a) T1 image (#0045). (b) T2 image (#0045). (c) Reference change map. (d) Ours. (e) IFN. (f) DASNet. (g) CD-UNet++. (h) FC-Siam-Conc. (i) FC-Siam-Diff. (j) FC-EF.

For the LEVID-CD dataset, the experimental results and visual comparisons with the seven SOTA methods (FC-EF [39], FC-Siam-Diff [39], FC-Siam-Conc [39], CD-UNet++ [42], DASNet [45], IFN [46], and GANs [43]) are shown in Table 3 (some of the experimental results were obtained from [47]) and Figures 8to 11.

In this experiment, four typical change areas, single-object change, more-object change, much-more-object change, and dense-object change, were selected to evaluate the performance of the proposed method. As can be seen in Figures 8to 11, the proposed method achieved the best performance. As the number of objects for CD increases, the proposed method maintains good recognition performance. The proposed method can generate an accurate CM even if the size or number of objects changes.

Meanwhile, the quantitative results on the four quantitative evaluation metrics listed in Table 3 show that the proposed method achieved excellent performance in terms of precision, recall, *F*_1_-score, IoU, and OA. Compared with the seven SOTA CD methods, the precision, recall, *F*_1_-score, IoU, and OA of the proposed method were 0.877, 0.889, 0.873, 0.852, and 0.991, respectively, being 0.031, 0.006, 0.012, 0.005, and 0.004 higher than their counterparts for the GANs method (the second-best method).

## 5. Conclusions

In this paper, we proposed a CD method for multisource remote sensing images using a coupled dictionary and deep learning. To reveal the coupled relationship between multisource remote sensing images, a coupled dictionary module was proposed. This module maps multisource remote sensing images from low-dimensional to high-dimensional space to calculate the reconstruction coefficient. The changing image blocks will have different reconstruction coefficients, while unchanging image blocks will have similar reconstruction coefficients. To more accurately describe the degree of change in an object, we constructed a new image block for reconstruction loss in a differential feature discriminant network, which measures the change degree of each image block pair by considering true/false discrimination loss, classification loss, and cross-entropy loss. Compared with other CD methods, the proposed method can effectively alleviate the uncertainty of edge pixel CD and realize satisfactory results. In the future, we plan to focus on CD for large remote sensing images and extend the proposed method to practical applications.

The method that we proposed has its limitations. The location where has changed in multisource remote sensing images can be detected by the proposed method. But what has changed in multisource remote sensing images cannot be detected. To address this problem, hyperspectral images containing a lot of spectral information can be used to detect what has changed. In addition, cloud and fog occlusion in remote sensing image can also affect change detection. Therefore, it is necessary to preprocess remote sensing images before using the proposed method for change detection. These limitations indicate that although a highly efficient change detection method is proposed, it is still a challenging task to generalize the method to practical production, and this will be the direction of our future efforts.

## Figures and Tables

**Table 1:** Ablation experiments results for the LEVID-CD dataset.

Methods	Precision	Recall	*F*_1_-score	IoU	OA
Sparse representation	0.724	0.715 8	0.711	0.718	0.854
+ CDL	0.796	0.785	0.801	0.763	0.895
+ CDL + DFE	0.812	0.806	0.828	0.783	0.913
+ CDL + DFE + *L*_*G*_	0.883	0.861	0.872	0.860	0.985

**Table 2:** Comparison experiment results of CDD.

Methods	Precision	Recall	*F*_1_-score	IoU	OA
FC-EF	0.815	0.761	0.771	0.698	0.941
FCN-PP	0.826	0.805	0.871	0.737	0.954
FC-Siam-Conc	0.844	0.825	0.825	0.751	0.957
FC-Siam-Diff	0.858	0.836	0.837	0.771	0.958
CDNet	0.827	0.817	0.822	0.797	0.964
CD-UNet++	0.876	0.859	0.868	0.828	0.967
GANs	0.881	0.856	0.865	0.847	0.974
Proposed method	0.883	0.861	0.872	0.860	0.985

**Table 3:** Comparison experiment results of the LEVID-CD dataset.

Methods	Precision	Recall	*F*_1_-score	IoU	OA
FC-EF	0.489	0.859	0.623	0.790	0.938
FC-Siam-Diff	0.542	0.732	0.631	0.793	0.960
FC-Siam-Conc	0.604	0.766	0.682	0.822	0.963
CD-UNet++	0.790	0.841	0.815	0.828	0.980 5
DASNet	0.774	0.899	0.828	0.836	0.980 6
IFN	0.796	0.880	0.836	0.839	0.982
GANS	0.846	0.883	0.861	0.847	0.987
Proposed method	0.877	0.889	0.873	0.852	0.991

## Data Availability

The underlying data supporting the results of our study can be found in [36] and [37].
